# A randomised controlled trial among cleaners-Effects on strength, balance and kinesiophobia

**DOI:** 10.1186/1471-2458-11-776

**Published:** 2011-10-10

**Authors:** Marie Birk Jørgensen, John Ektor-Andersen, Gisela Sjøgaard, Andreas Holtermann, Karen Søgaard

**Affiliations:** 1 The National Research Centre for the Working Environment, Lersø Parkallé 105, 2100 Copenhagen, Denmark; 2 Department of Exercise and Sports Sciences, University of Copenhagen, Copenhagen, Denmark; 3 Clinical Alchohol Research Lund University, Lund University, Lund, Sweden; 4 Institute of Sports Science and Clinical Biomechanics, University of Southern Denmark, Odense, Denmark

## Abstract

**Background:**

Cleaners constitute a job group with poor health and low socioeconomic resources. Therefore, there is a great need for scientifically documented health promoting initiatives for cleaners. However, both workplace initiatives and high quality intervention studies are lacking. The aim of this study was to evaluate the effects of a 3-month workplace trial with interventions to improve physical or cognitive behavioural resources among cleaners.

**Methods:**

A cluster randomised controlled trial was conducted among 294 female cleaners from 9 workplaces. The participants were allocated to three groups: Physical coordination training (PCT, n = 95), Cognitive behavioural theory-based training (CBTr, n = 99) and Reference group (REF, n = 100). Interventions were conducted during work hours for an average of 1 hour/week. Muscle strength was measured by maximal voluntary contractions in trunk/extension, and shoulder abduction/elevation. Postural balance was measured on a force platform. Kinesiophobia was measured with Tampa Scale for Kinesiophobia. Test and questionnaires were completed at baseline and at 3-month follow-up and analyses followed the intention-to-treat (ITT) principle with last observation carried forward in case of missing data at follow-up. Reports and analyses are given on true observations as well.

**Results:**

ITT-analyses revealed that PCT improved strength of the trunk (p < .05) and postural balance (p < .05) compared to CBTr and REF. Based on true observations the strength and balance improvements corresponded to ~20% and ~16%, respectively. ITT-analyses showed that CBTr reduced kinesiophobia compared to PCT and REF (p < .05). Based on true observations, the improvement corresponded to a ~16% improvement.

**Conclusion:**

This workplace-based intervention study including PCT and CBTr among cleaners improved strength and postural balance from PCT, and kinesiophobia from CBTr. The improved strength, postural balance and kinesiophobia may improve the cleaners' tolerance for high physical work demands. Future studies should investigate the potential in the combination of PCT and CBTr in a workplace intervention.

**Trial registration:**

Current controlled trials ISRCTN96241850

## Background

Cleaners defined as people whose main job task is cleaning work (janitors, custodians, cleaning assistants) constitute a job group with poor health and low socioeconomic resources [1-4]. Therefore, there is a great need for scientifically documented health promoting initiatives for cleaners. However, high quality intervention studies are lacking [5].

Ergonomic interventions have been conducted to prevent deterioration of cleaners' health by reducing the physical workload [1,6,7]. However, due to a high degree of work intensification, and the fact that some cleaning tasks are currently no further changeable, cleaning will remain a physically heavy work [4]. Another preventive strategy may be to improve the tolerance to the physical workload of the cleaner by enhancing the physical resources or the ability to cope with musculoskeletal pain [5]. High physical resources have previously been shown important for tolerating high physical work demands [8]. Moreover, pain-related fear of movement (kinesiophobia) has been shown to be a stronger predictor for future disability than pain itself [9].

Intervention programmes with physical training to improve strength have been successfully tested among office workers [10,11]. However, no previous high quality studies have investigated the effects of physical training on physical resources among cleaners. Cleaning work frequently involves pushing and pulling, standing, walking and squatting and job tasks with bent back, elevated hands and twisted body [2]. Therefore, interventions to improve muscle strength and postural control such as coordination training may be particularly relevant for preventing deterioration in this job group.

Several intervention studies have successfully focused on improving patients' pain coping abilities through programmes with cognitive behavioural therapy (CBT) as secondary prevention [12]. However, CBT has seldom been applied for primary prevention of health impairments or deterioration among working populations [13]. Because cleaners have high physical work demands and high prevalence of pain [1], pain-related fear of movement may be of particular importance for the risk of functional deterioration in this job group. Therefore, interventions to reduce pain-related fear of movement may be especially relevant for this job group.

The main aim was to test if workplace intervention trials with physically intensive strength coordination training (PCT) can improve muscle strength and postural control and if cognitive behavioural theory-based training (CBTr) can reduce kinesiophobia. Therefore, a randomised controlled trial with a PCT programme and a CBTr programme specifically tailored for cleaners was conducted on 9 cleaning workplaces in Denmark.

## Methods

The study is a part of the previously described FINALE (Frame for INterventions for preserved work Ability; Long term Effect) programme [5] aiming at investigating preventive initiatives against deterioration among work groups with high physical work demands. The study was a cluster randomised controlled intervention conducted at 9 cleaning workplaces in Denmark. Approval was received from the local ethics committee (H-C-2007-0033). The primary outcomes for this clinical trial are work ability and sickness absence. The current paper evaluates the mediating variables at 3-months follow-up. Trial effects on the primary outcomes are reported in Jorgensen et al. (submitted). Other publications in relation to the trial can be found by visiting http://www.controlled-trials.com and search for our unique trial registration number: ISRCTN96241850.

### Recruitment procedure

Participants were recruited from cleaning work places in the urban, rural and metropolitan regions of Denmark. Recruited participants were required to be employed at least 20 hours/week at the workplace, and primarily work during day hours. Their main work task had to be cleaning, but their job could also involve other service tasks such as washing, kitchen work or attending patients. All eligible employees (N = 758) were invited to an information meeting during their working hours, asked to answer a screening questionnaire, and to give consent if willing to enrol in the study. For employees who did not attend the information meeting, managers subsequently handed them written information on the project and screening questionnaires with a stamped addressed envelope. Among the eligible employees, 394 consented to participate, and these were invited to answer a questionnaire. Among the consenters, 363 (48%) entered the intervention study. A large fraction of immigrants are employed at cleaning workplaces in Denmark, but Danish language skills are required. Therefore, all written and oral communication was conducted in Danish. Language and reading difficulties were dealt with through guidance from the research personnel or collegial assistance. Further details of the recruitment procedure has previously been reported [14].

### Randomisation procedure

For the cluster randomisation procedure, each workplace was considered a stratum. Clusters depended on work teams where possible or were made up from groups either in which employees had lunch, groups where they worked in close proximity to each other, or groups who reported to the same manager. Clusters were matched on sex, age and job seniority. The randomisation was made by lot conducted by blinded staff. To obtain a homogeneous group and due to the high proportion of females in the job group, the 69 males were excluded from further analyses in the present study (PCT = 25 males, CBTr = 22 males, REF = 22 males).

### Design

The female cleaners were randomised into PCT (n = 95), CBTr (n = 99) or REF (n = 100). Both data collection and interventions were conducted during the cleaners' working hours. Questionnaires were collected and physical tests conducted at baseline and after three months intervention.

### Interventions

The PCT was instructor-guided and offered 3 times a week during 12 weeks in sessions of 20 minutes duration at the workplace during working hours. Six intensive physical coordination exercises with 2-4 levels of progression were performed. The exercises were four point kneeling, prone plank, bridge, vertical plank, bodyblade and horizontal side support and are described in detail elsewhere [15]. Each training session began with a short warm up consisting of ballistic stretching. During the first 1 or 2 weeks, only 3-4 exercises were presented at each session until the performance was technically correct. Thereafter each exercise was performed 2 times 25 seconds in a circuit programme. The exercises all involved a high demand of coordination and were performed at the maximal level of progression, challenging the performer optimally during the 25 seconds with a work load corresponding to 60-80% of their maximal voluntary muscle strength. One or two instructors supervised the participants' performances and offered corrections when necessary.

The CBTr was instructor-guided and offered every second week during 12 weeks in two-hour sessions at the workplace during working hours. The programme was a modified version of the programme developed by Linton [16,17]. With respect to the hypothesis of this paper, the purpose of the programme was to reduce kinesiophobia and thereby diminish negative effects of kinesiophobia. Therefore, the programme focused on improving the participants' understanding of pain, the experience of pain, and the anticipation of pain by performing cognitive exercises on how physical activity not necessarily leads to pain. Cognitive and behavioural exercises were performed to train the ability to function despite pain (i.e. pain coping, increasing health behaviour, adapt skills to daily life). Moreover, the positive long-term effects from appropriate pain coping were discussed [17]. Every session was planned with the same general outline: feedback on homework from previous session, introduction to today's theme of the session, a short lecture on the theme, problem-solving exercises and training of new skills (i.e. applied relaxation training) [18]. To enable the participants to train the applied relaxation programme during the workday, they received an mp3 with the necessary instructions.

### Measurements and analyses

#### Physical capacity

Isometric muscle strength was tested in maximal voluntary contractions (MVC) with validated measures on trunk extension and flexion, and shoulder elevation [19]. For shoulder elevation and abduction strength, the participant was sitting upright in a height-adjustable chair with Bofors dynamometers 1 cm medial to the lateral edge of the acromion of each shoulder and 1 cm proximal from the olecranon of the elbow joints, respectively. For the trunk extension and flexion, the participant was standing in an upright position with a strap attached to a strain gauge dynamometer around the shoulders at the level of deltoid insertion. For MVC of the back extensor muscles, the subject faced the dynamometer with the pelvis against a plate placed with the upper edge aligned with the iliac crest of the subject. Correspondingly, for MVC of the abdominal muscles, the subject was placed with the back towards the dynamometer and the pelvis against the plate. The vertical distance between the L4/L5 level and the middle of the strap was measured for torque calculation. In these isometric positions, the cleaner was instructed to gradually build up force over 5 sec, then to keep maximal force for about 2 sec, and finally to lower the force slowly to zero. This MVC was performed at least three times for each exercise. If the third recording was more than 5% higher than the previous two recordings, a fourth test was performed, with a maximum number of five tests. Strong standardized verbal encouragement was given during all trials. Cleaners were excluded from this test in case of self-reported or measured high blood pressure, angina pectoris, previous herniated disc or use of heart or lung medicine. Furthermore, the cleaners were asked about musculoskeletal symptoms within the last 7 days. In case of a positive answer, they were asked if they felt considerable pain in this specific body region on the test day. If so, they were excluded from the muscle strength measurement affecting this body region. A high proportion of the cleaners was in pain or had elevated blood pressure, as previously reported [20]. All cleaners were still invited to the randomised study.

A 30 sec balance test was performed in an undisturbed environment. The participants were encouraged to take a break in between repetitions of the test whenever they felt their attention to the task decreased. A crew of trained experimenters conducted and gave standardized instructions to each test. Participants stood barefooted on a force platform (AMTI, platform type OR6-7-1000, amplifier type MSA-6) and were instructed to "stand as still as possible". During the balance test, indications of test progression (10 sec, 20 sec, test ends) were verbally informed to the participant from the experimenter. If the participant lost balance during tests and moved arms or feet from the starting position, a new trial was recorded. Two tests were performed in the Romberg position (standing with feet together, heel-to-heel and toe-to-toe) with open and closed eyes [21]. The participants stood with their arms folded across their chest and their feet parallel to the y-axis of the platform. In the test with open eyes, participants were instructed to look at the black spot. The test was performed for 30 s and three trials were recorded for the test with closed eyes.

The force (*Fx, Fy *and *Fz*) and moment (*Mx, My *and *Mz*) signals were sampled at 125 Hz, and filtered (10 Hz 4^th ^order Butterworth zero-phase low-pass filter). The COP consisting of the AP and medio-lateral (ML) components ([*x_AP_, x_ML_*] = [*Mx/Fz, My/Fz*]) was calculated and decomposed into a rambling and trembling component. Subsequently, the 95% confidence ellipse areas (CEA) were calculated for the COP [22]. Participants were excluded from this specific test if reporting considerable pain, trauma (strain or overload) or physical restriction due to recent trauma in the neck, low back, hip, knee or ankle.

#### Kinesiophobia

Kinesiophobia is defined as "an irrational and debilitating fear of physical movement and activity resulting from a feeling of vulnerability to painful injure or (re)injury" [23]. Kinesiophobia is a marked or persistent fear that is often excessive or unreasonable, which is cued by the presence or anticipation of a pain-eliciting situation. Kinesiophobia is evident both among chronic pain patients [24] and in the general population with non-persistent pain [25]. Kinesiophobia is shown to precede avoidance-behaviour, which may have both physical and psychological health consequences [23]. Kinesiophobia was assessed by the questionnaire Tampa Scale for Kinesiophobia (TSK) [23]. A high value on the TSK indicates a high degree of kinesiophobia.

The TSK contains 17 items that are scored by 4-point Likert scales with scoring options ranging from 1 = "strongly disagree" to 4 = "strongly agree". The first 13 of the 17 items indicate increasing kinesiophobia with increasing scores and were phrased: "I'm afraid that I might injury myself if I exercise", "If I were to try to overcome it, my pain would increase", "My body is telling me I have something dangerously wrong", "People aren't taking my medical condition seriously enough", "My accident has put my body at risk for the rest of my life", "Pain always means I have injured my body", "I am afraid that I might injure myself accidentally", "Simply being careful that I do not make any unnecessary movements is the safest thing I can do to prevent my pain from worsening", "I wouldn't have this much pain if there weren't something potentially dangerous going on in my body", "Pain lets me know when to stop exercising so that I don't injure myself", "It's really not safe for a person with a condition like mine to be physically active", "I can't do all the things normal people do because it's too easy for me to get injured", "No one should have to exercise when he/she is in pain". Following these, four items with inverse structure were posed separately. The phrasing of these were: "My pain would probably be relieved if I were to exercise", "Just because something aggravates my pain does not mean it is dangerous ", "Although my condition is painful, I would be better off if I were physically active", "Even though something is causing me a lot of pain, I don't think it's actually dangerous". The scorings from the last four items were inverted before calculating the sum-score, thus ranging from 17-68. Some of the cleaners failed to answer the questionnaire, explaining the missing observation displayed from the number of responders in table 1.

**Table 1:** Baseline characteristics stratified on intervention groups

	Physical coordination training (N = 95)			Cognitive behavioural training (N = 99)			Reference group (N = 100)		
*Baseline*	n	Mean	SD	n	mean	SD	n	mean	SD
Age (years)	95	44	9.1	99	46	8.9	100	45	9.6
Height (cm) ┼	89	160	7.1	95	161	7.7	89	163	7.8
Weight (kg)	87	73	14.5	95	72	17.1	87	73	14.5
BMI (kg/m	87	28	5.1	95	28	5.9	87	28	5.0
Fat %	85	36	7.5	95	35	8.2	82	35	8.3
Job seniority (years)	74	9.4	9.1	83	9.9	8.1	78	10.3	9.6
Immigrants (%)	89	50.6	■	95	46.3	■	97	45.9	■
Muscle strength									
Shoulder elevation (Nm)	44	53.0	19.8	45	52.2	19.6	45	56.7	20.0
Shoulder abduction (Nm)	43	31.7	11.4	46	31.5	11.9	41	30.1	6.7
Trunk flexion (Nm)	48	93.2	29.5	47	103.7	44.9	51	101.5	29.9
Trunk extension (Nm)	45	81.4	28.9	43	89.2	30.5	48	88.7	37.1
Balance									
95% confidence ellipse (mm2)	82	822.5	506.5	88	771.1	529.4	81	787.1	500.9
Rambling (mm2)	82	495.7	324.7	88	442.9	295.6	81	453.8	274.7
Trembling (mm2)	82	155.4	108.8	88	169.9	160.4	81	169.1	139.0
Kinesiophobia (Index TSK17)	77	34.3	8.5	85	32.0	8.6	80	34.4	9.9
*Intention-to-treat baseline*									
Muscle strength									
Shoulder elevation (Nm)	47	51.8	19.8	45	52.2	19.6	47	57.1	19.7
Shoulder abduction (Nm)	47	33.6	13.1	47	31.8	12.0	44	30.4	6.9
Trunk flexion (Nm)	51	93.7	29.0	47	103.7	44.9	52	101.1	29.7
Trunk extension (Nm)	48	82.0	28.2	44	90.3	31.0	49	88.0	37.0
Balance									
95% confidence ellipse (mm2)	87	835.4	501.0	91	756.9	526.6	85	804.9	514.1
Rambling (mm2)	87	506.6	327.5	91	435.4	293.8	85	463.9	278.5
Trembling (mm2)	87	156.3	107.6	91	166.2	159.1	85	174.7	153.5
Kinesiophobia (Index TSK17)	82	34.9	8.6	91	32.6	9.0	83	34.6	9.7
*True observations baseline*									
Muscle strength									
Shoulder elevation (Nm)	26	52.0	19.9	23	51.4	19.3	24	55.9	21.2
Shoulder abduction (Nm)	23	31.8	9.2	24	29.7	9.0	22	29.2	6.5
Trunk flexion (Nm)	31	89.8	26.4	25	105.0	48.4	26	99.2	26.2
Trunk extension (Nm)*	28	76.7	26.1	23	87.3	30.8	25	99.9	37.0
Balance									
95% confidence ellipse (mm2)	50	856.6	540.9	45	733.0	409.7	54	824.2	579.2
Rambling (mm2)	50	517.4	333.6	45	430.2	268.9	54	470.6	314.7
Trembling (mm2)	50	160.5	117.1	45	155.7	106.8	54	180.4	159.5
Kinesiophobia (Index TSK17)	38	31.4	8.2	42	30.5	8.6	53	32.9	9.6

The *Baseline *reflects all of the female cleaners with observations at baseline, *Intention to treat baseline *reflects all of the female cleaners with observations at baseline including those with missing observations, for whom values were carried backwards from follow up, and *True observations baseline *reflect those cleaners with observations at both baseline and follow-up. N = number of participants, n = number of observations, Nm = Newton meter, SD = Standard deviation

### Statistics

To determine if differences between the three groups had happened by chance in the randomisation, descriptive data regarding the variables age, height, weight, body mass index, fat %, job seniority, distribution of immigrant and native cleaners, muscle strength, balance and kinseiophobia were reported. Since no differences appeared from these reports, the analyses on intervention effects were unadjusted. The effects of intervention on muscle strength, balance and kinesiophobia were evaluated in an intention-to-treat analysis using one-way analysis of variance (ANOVA) on the difference between baseline and follow-up, followed by Bonferroni corrected post-hoc-tests when relevant. Due to missing observations, observations were carried backward and forward at baseline and follow-up, respectively, thereby avoiding bias of non-random drop-out [26,27]. In addition, to display the true observations and to avoid false negative results, data were analysed exhaustive by performing the same analysis procedure only with true observations on both outcome measures at baseline and follow-up. This procedure was termed true-observations analysis. Finally, to obtain further power to the dataset, if no difference between REF and the other intervention group existed, both analyses were further explored in an aggregated analysis, collapsing REF with the inefficient intervention. For example, the PCT intervention effect on muscle strength and balance was tested against CBTr and REF combined, and the CBTr intervention effect on kinesiophobia was tested against PCT and REF combined. IBM SPSS statistics version 19 was used for all statistical analyses.

## Results

### Employee flow

**Figure 1:**
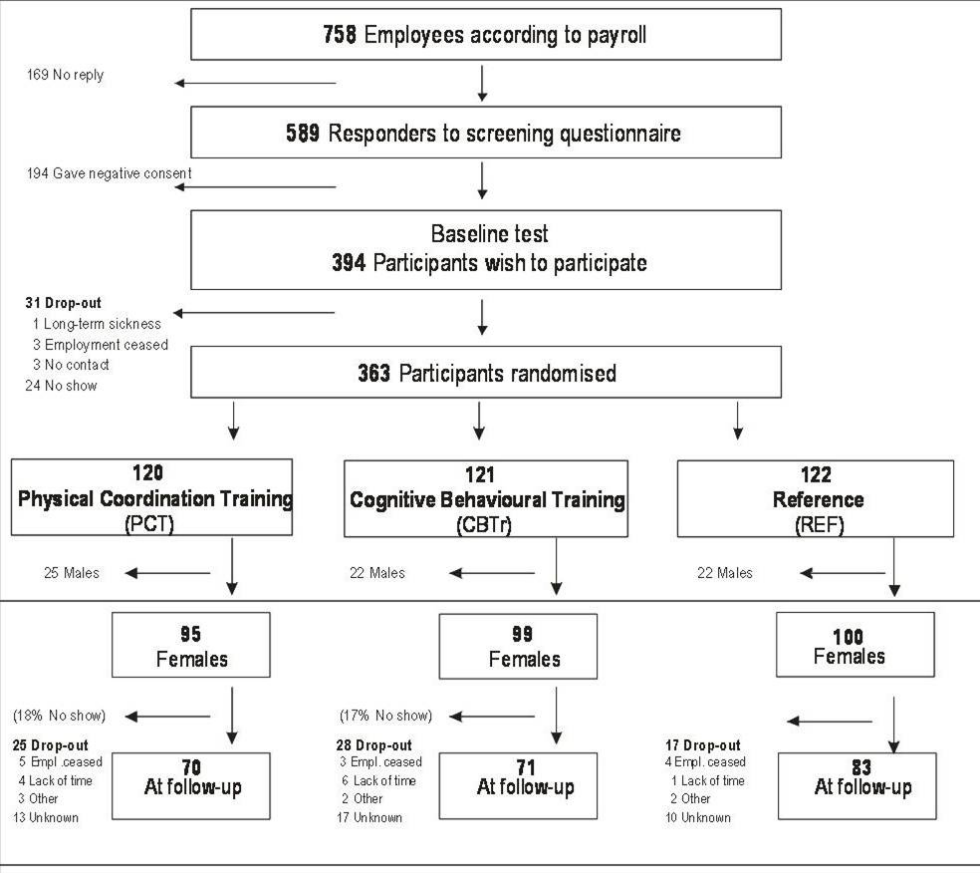
**Flow of participants through the intervention study**.

There were 33, 40 and 26 cleaners who dropped out of the study from PCT, CBTr and REF, respectively, as given in Figure 1. Those who dropped out did not attend the follow-up measurements. Seventeen and eighteen percent did not receive the intervention at any time in the PCT and CBTr, respectively. Mean adherence rates (including those with zero adherence) were 37% and 49% in PCT and CBTr, respectively.

### Baseline characteristics

Baseline characteristics of the participants in the three intervention groups are given in table 1. No differences between the three groups were found at baseline. Both mean for the participants at baseline, the intention-to-treat baseline populations (with missing variables carried backwards) and the true observations baseline population (those with outcome measures both at baseline and follow-up) are displayed. A large fraction of the study population was immigrants, as given in Table 1. Among the immigrants, 49 different ethnic identities were present with the 8 most prevalent being Turkey, Marcedonia, Pakistan, Thailand, Philippines, Morocco, India and Serbia, representing 70% of the immigrants in the study population. Previously, studies on the immigrant population in the current study has been published [20,28,29].

### Physical capacity

Intervention effects are shown in table 2 on both intension-to-treat basis and on true observations. PCT significantly improved trunk flexion muscle strength in comparison with CBTr and REF separately (p = .01 and .045) in intention-to-treat and in the aggregated analysis (p = .01). True observations revealed a ~20% improved trunk muscle strength in PCT. Changes in none of the other muscle strength measures were significantly different after the intervention. PCT improved balance in rambling in comparison with CBTr (p = .02) and there was a tendency to an improvement in comparison with REF alone (p = .07) in intention-to-treat. The PCT improvement was highly significant in the aggregated analysis (p = .004). True observations revealed a ~16% improvement in rambling in PCT. The improvement in 95% confidence ellipse showed a tendency to be better in the PCT compared to CBTr and REF (p = .087), and the difference was close to statistical significance in the aggregated analysis (p = .051). No change was found in trembling after the intervention. The analyses on the true observations provided similar results (table 2).

**Table 2:** Changes after the intervention on outcome variables stratified on intervention

	Physical coordination training (N = 95)			Cognitive behavioural training (N = 99)			Reference group (N = 100)			Aggregated analyses
	n	mean	SD	n	mean	SD	n	mean	SD	p
*Intention-to-treat*										
Muscle strength										
Shoulder elevation (Nm)	47	0.4	13.0	45	0.0	7.7	47	0.3	7.4	
Shoulder abduction (Nm)	47	2.4	6.0	47	0.9	4.6	44	2.1	7.4	
Trunk flexion (Nm)	51	11.7	24.1	47	-2.6	19.4	52	2.1	15.0	**
Trunk extension (Nm)	48	4.2	16.3	44	-2.2	17.6	49	2.5	18.6	
Balance										
95% confidence ellipse (mm2)	87	-69.7	295.4	91	28.2	265.9	85	-16.5	320.4	*
Rambling (mm2)	87	-48.4	189.6	91	28.9	171.9	85	17.1	203.1	**
Trembling (mm2)	87	-3.7	68.6	91	-3.8	55.6	85	-16.2	99.3	
Kinesiophobia (Index TSK17)	82	1.3	5.4	91	-1.1	4.7	83	0.1	5.8	*
*True observations*										
Muscle strength										
Shoulder elevation (Nm)	26	0.7	17.7	23	0.0	10.9	24	0.6	10.4	
Shoulder abduction (Nm)	23	4.8	7.8	24	1.8	6.4	22	4.2	10.2	
Trunk flexion (Nm)	31	19.3	28.6	25	-4.9	26.6	26	4.3	21.2	**
Trunk extension (Nm)*	28	7.2	21.0	23	-4.2	24.5	25	4.9	26.1	
Balance										
95% confidence ellipse (mm2)	50	-121.3	383.1	45	57.0	378.1	54	-25.9	403.1	┼
Rambling (mm2)	50	-84.2	245.0	45	58.3	242.3	54	26.9	255.1	**
Trembling (mm2)	50	-6.5	90.7	45	-7.7	79.4	54	-25.4	124.1	
Kinesiophobia (Index TSK17)	38	2.8	8.6	42	-2.6	6.7	53	0.1	7.92	**

*Intention to treat *reflects all the female cleaners enrolled to the study, who had an observation at baseline or at follow-up. In case of missing observations, values are carried backwards or forward from the available observation, leaving a delta-value of 0. *True observations *reflect those with observations at both baseline and follow-up. N = number of participants, n = number of observations, Nm = Newton meter, SD = Standard deviation, p = Level of significance, ┼ p < 0.1, * p < 0.05, ** p =/< 0.01. In aggregated analyses the non efficient intervention group is considered a reference and is merged with the reference group. E.g. for kinesiophobia cognitive behavioural training is tested against physical coordination training+reference and for all other outcomes physical coordination training is tested against cognitive behavioural training+reference. In the muscle strength test n is different due to the health-related exclusion (self-reported or measured high blood pressure, angina pectoris, previous herniated disc, use of heart or lung medicine or current considerable pain on the body regions specific to the test). n is limited in the balance test due to the health-related exclusion (considerable pain, trauma or physical restriction due to recent trauma in the neck, low back, hip, knee or ankle).

### Kinesiophobia

The TSK had a high internal consistency, with Chronbach's alpha of 0.80. The CBTr significantly reduced their kinesiophobia after the intervention in comparison with PCT (p = .012), but not with respect to REF (p = .457). However the difference was significantly different in the aggregated analysis, CBTr versus PCT and REF (p = .013). Furthermore, these differences were highly significant in the true observations analysis both against PCT (p = .008) and against PCT and REF aggregated (p = .01). The true observations revealed a ~16% reduced kinesiophobia in CBTr.

## Discussion

### Main findings

This 3-month randomised controlled trial among female cleaners from 9 workplaces in Denmark significantly improved their individual physical and cognitive behavioural resources. The PCT intervention improved trunk muscle strength and balance, and the CBTr intervention reduced kinesiophobia. In the following, implications and perspectives on the improvements of these resources for the prevention of deterioration among cleaners will be discussed.

### Comparison with other studies

Our study is the first RCT among workers with high physical work demands demonstrating a workplace training intervention to improve muscle strength in an ITT-analysis. It is well recognized that the dose of a work task is relative to the capacity of the performing worker [30,31]. Therefore, the relative physical work exposure on the musculoskeletal system of the worker can be considered reduced when strength improvements are obtained. Generally, the strength level of the cleaners in the current study was comparable to reports from previous studies with a representative sample of the Danish population [32] and a sample of Danish cleaners [8], although trunk strength was below the previous reports. High muscle strength has been shown to characterize senior (> 8 years) cleaners without muscle pain in comparison with cleaners with similar seniority with muscle pain [8]. Therefore, the increased muscle strength from the PCT may improve the cleaners' tolerance for high work loads and possibly reduce the risk for deterioration, i.e. musculoskeletal disorders.

The PCT was tailored to improve both strength and coordination of the cleaners. Accordingly, the PCT resulted in an improved postural balance. The PCT included training of the *bracing *manoeuvre, which produces a global co-activation of the muscles of the abdominal wall [33]. In our study, the only instruction that was given during the balance test was "stand as still as possible" and the test leader was blinded to the randomisation. Therefore, the improved balance may imply that the cleaners in PCT were able to transfer and use the improved strength and coordination of abdominal wall muscles in tasks not related to the training. Some studies suggest that poor stabilisation may predispose injury and musculoskeletal disorders [34-36]. Thus, the improvements in strength and balance in PCT may prevent deterioration of health among female cleaners in the longer term.

CBTr was shown to decrease kinesiophobia compared to both REF and PCT. Pain-related fear of movement is closely related to measures of disability and catastrophising [9,37-39]. Furthermore, kinesiophobia is shown to predict long-term recovery from pain-related functional disability among males with chronic non-specific low back pain [40] and improved kinesiophobia among work-disabled pain patients have shown positive effects on return to work [41]. Thus, reductions in kinesiophobia may reflect reduced pain-related fear of movements related to work tasks.

Baseline values of kinesiophobia among the working cleaners were on average 32-34. These values are comparable to a sample of the Dutch general population [25] and less than the average of 38-40 reported from patients seeking care due to musculoskeletal pain [40,42,43]. Although baseline levels were lower in the current study population, significant improvements were still found. This introduces CBTr as a possible valuable prevention strategy to reduce kinesiophobia in workplace interventions as well as in rehabilitation.

No effects on kinesiophobia, strength and balance were seen across the interventions. This finding indicates that contamination was successfully avoided by the cluster randomisation. However, five out of seven measures of strength and balance were numerically impaired in the CBTr-group, and kinesiophobia was numerically increased in the PCT-group. These changes were not significantly different from REF, and conclusions can not be drawn on these aspects. However, for improvements to be fully reflected in the ability to tolerate high physical work demands, interventions to counteract the reduced resources and thus improve both physical and cognitive-behavioural resources would probably be optimal. We therefore suggest that future interventions should integrate both PCT and CBTr in one initiative in the prevention on physical deterioration among workers. Future research is needed to verify this recommendation.

### Strengths and limitations

This study tested new approaches to prevention of health deterioration among 294 female cleaners from nine representative workplaces [14]. The interventions were thoroughly developed and tailored to the specific job group of cleaners. Although adherence rates were rather low, they were not lower than intervention studies in similar job groups [44]. However, the development of the interventions primarily built on a theoretical rationale derived from efficacy studies. Efficacy studies differ from effectiveness studies by being conducted in a context that gives optimal conditions for implementation [45]. Efficacy is necessary to, but not sufficient for effectiveness [45] and implementation is suggested to be thought of as interacting with the efficacy to determine effectiveness [46]. Thus in an effectiveness study, implementation plays an important role in obtaining results. In the current study, two specific efforts were made to support implementation of the interventions. First, workplaces adopting this intervention study were obliged by contract to provide time for the intervention during working hours. Second, each training session was guided by an instructor to personalise the interventions. Although adaption to the workplace setting was performed and pilot studies conducted, the practical rationale behind the interventions could have gained strength, if they had derived from feasibility studies among cleaners. Both inadequate efficacy as well as implementation is a possible reason for the lack of effect in some of the strength parameters. Thus, further efforts to improve implementation and adherence rates of workplace intervention studies in job groups with low socio-economic resources and among workers with low influence on work schedules should be implemented in future study designs.

By the conservative intention-to-treat analyses (with forward and backward carrying of missing observations), a tendency to underestimate the variances appears. It should be mentioned though, that the analyses reported in the results section of this paper follow the standards of the consort statement [26] and are conservatively designed to false positive finding due to avoid bias associated with non-random drop-out. Even with the relatively large drop-out, intention-to-treat analyses were able to reveal significant intervention effects, supporting our hypotheses of the interventions. Nevertheless, given the large standard deviations shown in table 2 it is likely that this study suffers from impaired power and some false negative results may be evident.

A non-significant increase in kinesiophobia was seen in the PCT-group. Since the increase was non-significant, it cannot be ruled out, that it happened by chance. However, according to the fear-avoidance theory of pain, one reaction to an expected painful stimulus may be avoidance behaviour [47]. That is, the individual has certain expectations on the painful consequences of an activity, which lead to avoiding the activity. It is well known, that physical training in itself can introduce an acute pain response [48]. Thus a confirmation of the fearful expectation to physical training may exacerbate the fear of movement and result in the increased kineisophobia seen in the current study. Graded activity has been suggested as treatment method for pain patients with high kinesiophobia. With graded activity, loads are introduced gradually and thereby producing disconfirmations of expectations of pain and harm and actual consequences of the activity [47]. In the PCT, exercise intensity was increased gradually and instructors carefully informed the participants that some pain and soreness could be experienced after training. However, no cognitive-behavioural or operant exercises were included. Actually, this was avoided to reduce overlapping interventions. In spite of the insignificance, the numerical increase in kinesiophobia in PCT may encourage that future training interventions corporate such kinesiophobia preventive exercises prior to or concurrent with the training.

## Conclusion

The main finding of this randomised controlled trial was that female cleaners improved their physical and cognitive-behavioural (psychological) resources after three months interventions. The PCT-intervention improved trunk flexion strength and postural balance. The CBTr-intervention reduced kinesiophobia, when compared with REF and PCT. This study is the first to reveal such improvements among workers with high physical demands. Indications were found for the increased potential of combining the PCT and CBTr interventions in future workplace interventions among work groups with high physical work demands and high prevalence of pain.

## List of abbreviations

PCT: Physical coordination training; CBTr: Cognitive behavioural theory-based training; REF: Reference group; MVC: Maximal Voluntary Contraction; ITT: Intention to treat

## Competing interests

The authors declare that they have no competing interests.

## Authors' contributions

MBJ, KS: Overall design. MBJ, KS, GS: Design of the PCT-intervention. MBJ, KS, JEA: Design of the CBTr-intervention. MBJ, KS, AHO: Conduct of data analyses. MBJ: First drafting of the manuscript. All authors read and approved the final manuscript.

## Pre-publication history

The pre-publication history for this paper can be accessed here:

http://www.biomedcentral.com/1471-2458/11/776/prepub
